# Platelets in Amyloidogenic Mice Are Activated and Invade the Brain

**DOI:** 10.3389/fnins.2020.00129

**Published:** 2020-03-03

**Authors:** Kathrin M. Kniewallner, Diana M. Bessa de Sousa, Michael S. Unger, Heike Mrowetz, Ludwig Aigner

**Affiliations:** ^1^Institute of Molecular Regenerative Medicine, Paracelsus Medical University, Salzburg, Austria; ^2^Spinal Cord Injury and Tissue Regeneration Center Salzburg (SCI-TReCS), Paracelsus Medical University, Salzburg, Austria; ^3^Austrian Cluster for Tissue Regeneration, Vienna, Austria

**Keywords:** platelets, Alzheimer’s disease, aging, vascular pathology, astrocytes

## Abstract

Alzheimer’s disease (AD) is a neurodegenerative disease with a complex and not fully understood pathogenesis. Besides brain-intrinsic hallmarks such as abnormal deposition of harmful proteins, i.e., amyloid beta in plaques and hyperphosphorylated Tau in neurofibrillary tangles, blood-derived elements, in particular, platelets have been discussed to be involved in AD pathogenesis. The underlying mechanisms, however, are rather unexplored. Here, we investigate a potential role of platelets in an AD transgenic animal model with severe amyloid plaque formation, the APP-PS1 transgenic mice, and analyzed the presence, spatial location and activation status of platelets within the brain. In APP-PS1 mice, a higher number of platelets were located within the brain parenchyma, i.e., outside the cerebral blood vessels compared to WT controls. Such platelets were activated according to the expression of the platelet activation marker CD62P and to morphological hallmarks such as membrane protrusions. In the brain, platelets were in close contact exclusively with astrocytes suggesting an interaction between these two cell types. In the bloodstream, although the percentage of activated platelets did not differ between transgenic and age-matched control animals, APP-PS1 blood-derived platelets showed remarkable ultrastructural peculiarities in platelet-specific organelles such as the open canalicular system (OCS). This work urges for further investigations on platelets and their yet unknown functional roles in the brain, which might go beyond AD pathogenesis and be relevant for various age-related neurodegenerative diseases.

## Introduction

Worldwide, approximately 50 million people live with dementia, a large number that is expected to triplicate by 2050 as the world population grows and the lifespan increases (International Alzheimer’s Disease [IAD], 2015). In about 60 to 70% of the patients, dementia is due to Alzheimer’s disease (AD), which makes AD not only the most common cause of dementia but also a major economic and social burden (International Alzheimer’s Disease [IAD], 2015; Van Cauwenberghe et al., 2015). Nevertheless, and more than a century after its first description, the exact etiology of AD pathology is still not understood (Piaceri et al., 2013). Moreover, there are currently no drugs available that can stop or reverse AD progression, and the approved therapeutics merely ameliorate symptomatology (Frozza et al., 2018).

Drug development in AD over the last decades repeatedly tried to target brain-intrinsic hallmarks of the pathology. These included neuroprotective approaches and attempts to reduce the load of harmful proteinaceous deposits in the brain, such as amyloid beta (Aβ) in plaques and hyperphosphorylated Tau in neurofibrillary tangles. So far, these approaches failed in the late clinical phases (Michael et al., 2019). Therefore, the amyloid cascade and the tau hypotheses as being causative in AD pathogenesis are increasingly questioned (De Strooper and Karran, 2016). In addition, novel concepts consider that the brain is not an isolated organ but is strongly influenced by systemic factors, which might contribute to brain degeneration or alternatively function as protective and/or self-regenerative mechanisms to compensate CNS damages. Along this line, increasing evidence illustrates that blood components are contributing to brain aging (Villeda et al., 2011; Smith et al., 2018) and that components present in young blood might be developed as therapeutics to treat AD (Middeldorp et al., 2016).

In the context of brain aging and neurodegeneration, blood-derived elements with therapeutic potential are platelets. Platelets have repeatedly been hypothesized to play a role in AD pathology (Li et al., 1998; Evin et al., 2003), in particular, in cerebral amyloid angiopathy (CAA) (Gowert et al., 2014; Jarre et al., 2014; Donner et al., 2016; Kniewallner et al., 2016, 2018). Platelets are small anuclear blood cells, which store a plethora of bioactive factors in specialized cytoplasmic compartments (Rumbaut and Thiagarajan, 2010). Upon activation, these factors are released to the extracellular space, allowing platelets to participate in diverse physiological and pathological processes (Rivera et al., 2016). In AD, vascular abnormalities, such as disrupted microvascular integrity or microbleedings, might trigger platelet activation in an attempt to restore vascular integrity. Once activated, platelets release among other substances Aβ peptides and several inflammatory mediators (e.g., IL-1β, CD40L, Thromboxane A2) (Arman et al., 2015; Canobbio et al., 2015; Heijnen and Korporaal, 2017; Humpel, 2017; Kniewallner et al., 2018). Thus, platelets might contribute to the deposition of Aβ peptides in the wall of cerebral blood vessels as well as to an inflammatory environment, which ultimately translates into vascular degeneration (Kniewallner et al., 2018). Whereas the involvement of platelets in the formation of cerebral amyloid angiopathy deposits has been a matter of extensive study, less is known concerning the general role of platelets in AD (Gowert et al., 2014).

Here, we investigated platelets in APP-PS1 transgenic mice with a severe amyloid plaque formation but no CAA pathology in the brain. We analyzed the spatial location of platelets in the brain with respect to their location within or outside blood vessels. We also studied blood-circulating platelets for their activation status by flow cytometry (FC) and analyzed their ultrastructure using electron microscopy.

## Materials and Methods

### Animals

Female and male APP Swedish PS1 dE9 mice (reviewed in Jankowsky et al., 2001) expressing a chimeric mouse/human mutant amyloid precursor protein (Mo/HuAPP695swe) and a mutant human presenilin 1 (PS1-dE9) both directed to CNS neurons under the prion protein promoter (Jackson Laboratory)^1^ were used. Mice were housed at the Paracelsus Medical University Salzburg in groups under standard conditions at a temperature of 22°C and a 12-h light/dark cycle with *ad libitum* access to standard food and water. Animal care, handling, genotyping, and experiments were approved by local ethical committees (BMWFW-66.019/0011-WF/V/3b/2016) and conducted accordingly to the Declaration of Helsinki. For brain histology, FC and transmission electron microscopy (TEM) mice were analyzed at 14 months of age. Age-matched non-transgenic mice derived from the breeding of APP-PS1 were used as control animals (WT).

### Tissue Processing

Mice were anesthetized with a solution of ketamine (20.5 mg/mL; Ketamidor; Richter Pharma), xylazine (5.36 mg/mL; Chanazine; Chanelle), and acepromacine (0.27 mg/mL; Vanastress 10 mg/mL; Vana GmbH) in 0.9% sodium chloride. In order to retain blood in the blood vessels, brains were extracted without being perfused and post-fixed with 4% paraformaldehyde in sodium phosphate buffer (0.1 M; pH = 7.4) overnight at 4°C. The brains were cryoprotected and transferred into 30% sucrose in 0.1 M sodium phosphate buffer (pH = 7.4). Sagittal sections of 40 μm were cut on dry ice, using a sliding microtome.

### Preparation of Washed Platelets

Upon thoracotomy, blood was collected by cardiac puncture using ethylenediaminetetraacetic acid (EDTA; 0.1 M; Promega) coated syringes. To further prevent coagulation, EDTA (0.1 M) was added to all blood samples. Anticoagulated blood samples were centrifuged at 200 × *g* for 20 min at room temperature (RT) to obtain platelet-rich plasma (PRP). PRP fractions were collected into new tubes, and 1 μM Prostaglandin E1 (Sigma-Aldrich #P55115-1MG) was added to avoid platelet aggregation, before centrifugation at 800 × *g*, for 20 min, RT. The supernatant was discarded and platelet pellets resuspended in Tyrode’s buffer (134 mM NaCl, 12 mM NaHCO_3_, 2.9 mM KCl, 1 mM MgCl_2_, 0.34 mM Na_2_HPO_4_, 10 mM Hepes solution). Before use, washed platelets in Tyrode’s buffer were allowed to rest for approximately 1 h, to minimize artificial platelet activation.

### Immunohistochemistry

Fluorescence immunohistochemistry of mouse tissue was performed on free-floating sections as previously described (Marschallinger et al., 2015; Unger et al., 2016, 2018). Antigen retrieval was performed depending on the used primary antibody by steaming the sections for 15–20 min in citrate buffer (pH = 6.0, Sigma). The following primary antibodies were used: rat anti-CD41 (1:300; Abcam), mouse anti-CD62P (1:300; Abcam), rabbit anti-collagen IV (1:50; Abcam), guinea pig anti-GFAP (1:500; Progen), goat anti-Iba1 (1:500; Abcam), guinea pig anti-NeuN (1:500; Millipore), rabbit anti-Olig2 (1:500; Merck), goat anti-PDGFRβ (1:100; R&D Systems). Incubation with primary antibodies occurred either overnight at RT or for 72 h at 4°C. After incubation with primary antibodies, sections were extensively washed in PBS and incubated for 3 h at RT in secondary antibodies (all at 1:1,000). The following secondary antibodies were used: donkey anti-goat Alexa Fluor 568, donkey anti-guinea pig Alexa Fluor 647, donkey anti-mouse Alexa Fluor 647, donkey anti-rabbit Alexa Fluor 488 and 568, donkey anti-rat Alexa Fluor 488, goat anti-rat Alexa Fluor 568 (all Invitrogen or Molecular Probes). For amyloid-β plaque staining, Thioflavin S (1 mg/mL; 1:625; Sigma-Aldrich) was added to the secondary antibody solution. Nucleus counterstaining was performed with 4′,6′-diamidino-2-phenylindole dihydrochloride hydrate (DAPI; 1 mg/mL; 1:2,000; Sigma-Aldrich).

### Electron Microscopy

Transmission electron microscopy (TEM) was used for morphological characterization of blood isolated platelets from APP-PS1 and WT age-matched controls (*n* = 3/group). Platelets dissolved in Tyrode’s buffer were fixed for 10 min with Cell Fix (BD Biosciences, 1:4). Upon centrifugation at 800 × *g*, for 20 min, at RT, platelet pellets were post-fixed in 2.5% glutaraldehyde (Agar Scientific), 4% PFA in phosphate buffer solution overnight. Upon centrifugation at 800 × *g* for 10 min, pelleted platelets were incubated in 1% osmium tetroxide (Electron Microscopy Sciences) in PBS Dulbecco (Merck) for 45 min in the dark. Following centrifugation at 800 × *g* for 10 min, pellets were washed twice in distilled water for 5 min. Between washes, platelets were centrifuged at 800 × *g* for 5 min. A third fixation step was carried out for 10 min in uranyl acetate replacement stain (1:10 in water; Electron Microscopy Sciences). Fixative solution was removed by centrifugation at 800 × *g* for 5 min, and platelets were washed as described previously. Washed pellets were dehydrated in a graded series of ethanol solutions, by incubation in ethanol 25% (5 min), 50% (5 min), 70% (5 min), 96% (5 min), and 100% (10 min). To finally complete dehydration, platelets were transferred to acetone (10 min), before being embedded in DurcupanTM ACM (four-component resin; Sigma-Aldrich). Resin curation was carried out at 37°C overnight and subsequently at 60°C for 72 h. Plastic blocks were trimmed to a trapezoid shape and sectioned using an ultramicrotome (Reichert-Jung Ultracut E, Austria). Semithin sections (0.5 μm) were generated with a glass knife and stained with 1% toluidine blue to confirm specimen exposure. After identification of stained cells, ultrathin sections (approximately 60 nm) were prepared using a diamond knife (Diatome Histo). Sections were flattened with chloroform and subsequently mounted on cooper grids (G75-Cu; Electron Microscopy Science), pre-coated with 0.2% Formvar. Transmission electron micrographs were obtained using a Leo 912 Omega transmission electron microscope (Carl Zeiss, Germany), at an operating voltage of 100 kV and magnification of 20,000×.

### Flow Cytometry Analysis

To investigate the basal activation level of circulating platelets in APP-PS1 and WT age-matched mice (*n* = 7/group), we performed two-color FC analysis in washed platelets. Before staining, a 50 μL aliquot of each sample was incubated with rat anti-mouse BD CD16/CD32 Fc Block TM (1:100; #553142; BD Biosciences) for 5 min at RT in FC buffer [2% bovine serum albumin (Sigma), 2 mM EDTA (Promega) in PBS Dulbecco (Merck)] to block unspecific binding sites. Thereafter, samples were stained with allophycocyanin (APC)-labeled anti-mouse CD41 (1:100; #133913; BioLegend) and fluorescein isothiocyanate (FITC)-labeled rat anti-mouse CD62P (1:100; #553744; BD Pharmingen) antibodies for 30 min in the dark. An aliquot of washed platelets incubated with thrombin from bovine plasma (0.25 U/mL; #1.12374; Sigma-Aldrich) and the respective antibodies, served as positive control for platelet activation. Additionally, isotype IgG staining was performed using FITC-rat IgG1λ isotype control (1:100; #553995; BD Pharmingen). Staining reaction was stopped with FC buffer, and samples were centrifuged at 800 × *g* for 10 min. Platelet pellets were resuspended in FC buffer and immediately analyzed with BD Accuri TM C6 Plus Flow Cytometer (BD Biosciences).

Platelet population was defined based on their pattern of forward and side scattering on a logarithmic scale and by positive staining for CD41, commonly used as a pan-platelet marker. Cell doublets were discriminated and excluded from the analysis. Per sample, at least 5,000 CD41^+^ single events were recorded. To determine the activation level of platelets, CD41^+^ cells were gated for CD62P^+^ expression. CD62P, also known as P-selectin, is a transmembrane glycoprotein mainly stored in platelet α-granules, which rapidly translocates onto the platelet surface upon activation (Rumbaut and Thiagarajan, 2010; Yun et al., 2016). The percentage of CD62P^+^ platelets was calculated relatively to the number of CD41^+^ platelets present in each sample. To compensate for non-specific immunofluorescence, the percentage of positive platelets detected in the isotype control staining for CD62P was subtracted from the percentage of CD62P^+^ platelets determined in each sample. For data presentation, FC plots were generated with Kaluza Analysis Software (Beckman Coulter).

### Microscopy, Visualization, and Image Processing

For qualitative and quantitative microscopic analysis of mouse tissue, confocal laser scanning microscopy was performed using a LSM 710 from Zeiss. Representative images were taken at 20×, 40×, and 63× magnification as confocal z-stack images and imported into the Imaris software (version 9.1.2, Bitplane) for 3D surface rendering and image analysis.

### Quantitative and Qualitative Analysis of Immunohistochemical Data

To analyze platelet distribution in the brain, four confocal images from the cortex and hippocampus of each animal (*n* = 6/group) were used for 3D modeling. Localization of platelets, visualized by CD41 staining, was assessed in relation to cerebral blood vessels, stained for collagen IV. The number of platelets located in the intra- and extraluminal spaces as well as their volume were determined. To study platelet activation *in vivo*, expression of CD62P was investigated in 100 CD41^+^ platelets present in the brain per animal, regardless of their location in relation to blood vessels (*n* = 6/group). To evaluate the occurrence of cellular interactions between platelets and Thioflavin S^+^ amyloid plaques (*n* = 2/group) and other cells of the neurovascular niche (*n* = 3/group), confocal Z-stacks from randomly selected visual fields of the hippocampus and cortex were employed for 3D surface rendering. Three-dimensional projection models were rotated around different angles in order to seek surface co-localizations. To quantify platelet–astrocyte interactions, four confocal images of the hippocampus were collected per animal (*n* = 3/group) and processed for 3D imaging in order to determine the number of CD41^+^ cells co-localizing with GFAP^+^ cells.

### Quantitative Analysis of Electron Microscopy Data

Per genotype, the surface and OCS areas of 90 randomly selected platelets (30 platelets/animal, three animals/genotype) were assessed using the Image J software (version 1.44p). The platelet surface area and OCS area of single platelets were analyzed, and the number of every analyzed platelet per genotype was used for statistical analysis. In order to determine the OCS area, the area of all white vacuole and tubular sections within the cell was measured. Relative OCS area was calculated accordingly to the formula:

$$\mathrm{Relative}\mathrm{OCS}\mathrm{Area}\left(\%\right)=\frac{\mathrm{OCS}\,\mathrm{measured}\,\mathrm{area}\times 100}{\mathrm{Platelet}\,\mathrm{measured}\,\mathrm{area}}$$

### Statistical Analysis

Statistical analysis was performed using GraphPad Prism (GraphPad Software, version 6). Data was tested for normality using the Shapiro–Wilk normality test. To compare values between two groups, unpaired Student’s *t*-test was used for normally distributed data and Mann–Whitney test for non-normally distributed data. For multiple comparisons between groups, two-way ANOVA with Tukey’s multiple comparison test was used. For statistical analysis of quantitative electron microscopy data, the ROUT method (*Q* = 1%) was used to identify outliers. Two outliers were identified and removed in the platelet area data set and one in the OCS relative area data set. Values of *p* < 0.0001 (^****^) were considered most significant, *p* < 0.01 (^∗∗^) highly significant, and *p* < 0.05 (^∗^) significant. Values were expressed as mean ± standard error of the mean (SEM).

## Results

### Platelets in APP-PS1 Mice Invade the Brain Parenchyma

**FIGURE 1:**
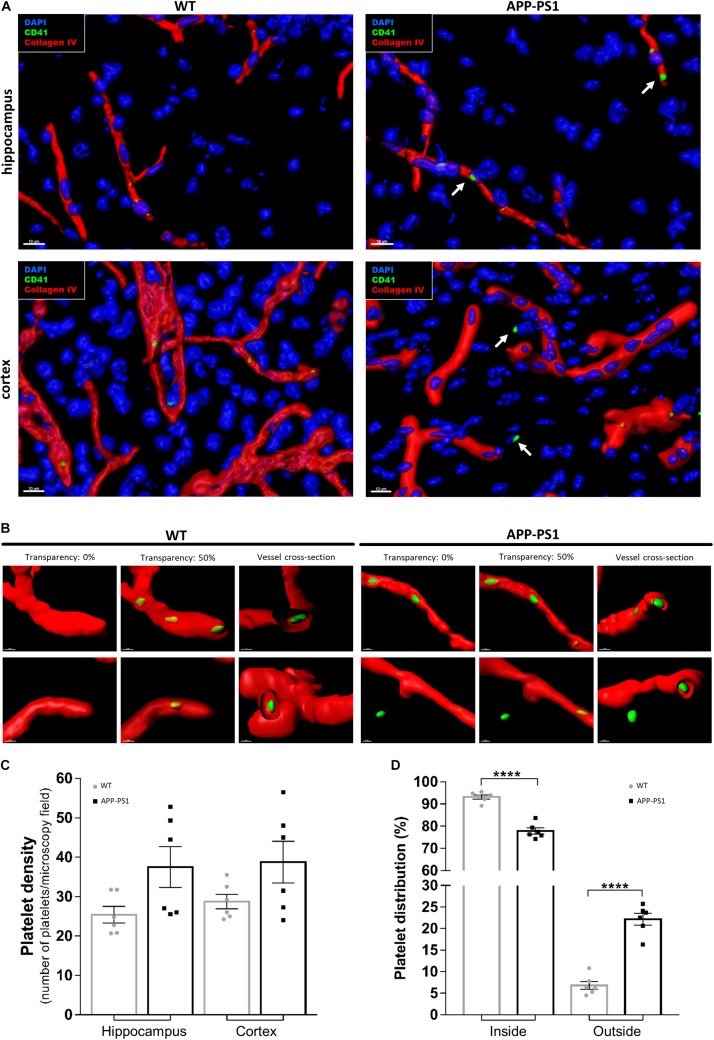
Immunohistochemical analysis of platelet distribution in the brain of APP-PS1 mice. Brain sections from APP-PS1 mice and age-matched WT controls were stained with collagen IV (red) for blood vessels and CD41 (green) for platelets. 4′,6′-diamidino-2-phenylindole dihydrochloride hydrate (DAPI; blue) was used as nucleus stain. Confocal microscopy images, from randomly selected hippocampal and cortical regions, were processed for 3D imaging. Platelets from WT and APP-PS1 mice were mainly located within the cerebral blood vessels **(A,B)**, whereas platelets from APP-PS1 mice were frequently seen in the brain parenchyma or at the blood vessel wall, suggesting extravasation **(A**—arrows, **B**—right**)**. Overall, APP-PS1 mice seem to have higher platelet densities in the hippocampus and cortex in comparison with WT controls **(C)**. Moreover, quantification of platelets located inside (blood vessel) and outside (parenchyma/vessel wall) cerebral blood vessels revealed that APP-PS1 mice have a significantly higher percentage of platelets outside the blood vessels compared to WT controls **(D)**. Data shown as mean ± SEM. Statistical analysis was performed by two-way ANOVA with Tukey’s multiple comparison test (*n* = 6/group; including WT: six females and APP-PS1: three females and three males); ****p < 0.0001. Scale: 10 μm **(A)** and 3 μm **(B)**.

First, we investigated platelet localization in the hippocampus and cortex of 14-month-old APP-PS1 transgenic mice and age-matched WT controls by immunohistochemical staining for CD41. We have selected APP-PS1 mice with the age of 14 months, as at this time point the animals present a full spectrum of pathology, showing severe amyloid plaque deposition in the hippocampus and cortex, high levels of microgliosis and astrogliosis, vascular alterations, and cognitive deficits (Jankowsky et al., 2004; Lalonde et al., 2005; Janota et al., 2015; Unger et al., 2016). To discriminate between platelets being completely inside or exposed to the outside of the blood vessels, we modeled the vessel structure in 3D with different levels of transparency, using the Imaris software. This allowed us to visualize platelets located within the vessels. In WT and APP-PS1 mice, CD41^+^ platelets were mainly located in the intraluminal space of blood vessels, or at least within the blood vessel’s basement membrane, which was outlined by collagen IV immunoreactivity (Figures 1A,B). Besides that, and more pronounced in APP-PS1 mice, platelets spread through the vessel wall, partially transposing into the extraluminal space (Figures 1A,B). Moreover, in APP-PS1 mice, platelets were also found completely outside the blood vessels in the brain parenchyma (Figures 1A,B), being in this case mostly located in the vicinity of a blood vessel (Figure 1B). Quantitative analysis in hippocampal and cortical brain regions revealed that APP-PS1 mice had a higher total number of platelets per brain section in comparison to WT controls (Figure 1C). As similar observations were made in the hippocampus and cortex (Figure 1C), we did no further discrimination between these two brain regions in subsequent analyses and pooled the data from both brain regions. A significantly higher percentage of platelets was found outside blood vessels in the extraluminal space in APP-PS1 compared to WT brains (22.18 ± 1.37% vs. 6.81 ± 0.92%), and vice versa; a significantly lower percentage of platelets were inside the vessels in APP-PS1 brains (77.83 ± 1.37% vs. 93.20 ± 0.92%) (Figure 1D).

### Characterization of Blood Circulating Platelets in APP-PS1 and WT Age-Matched Control Mice

**FIGURE 2:**
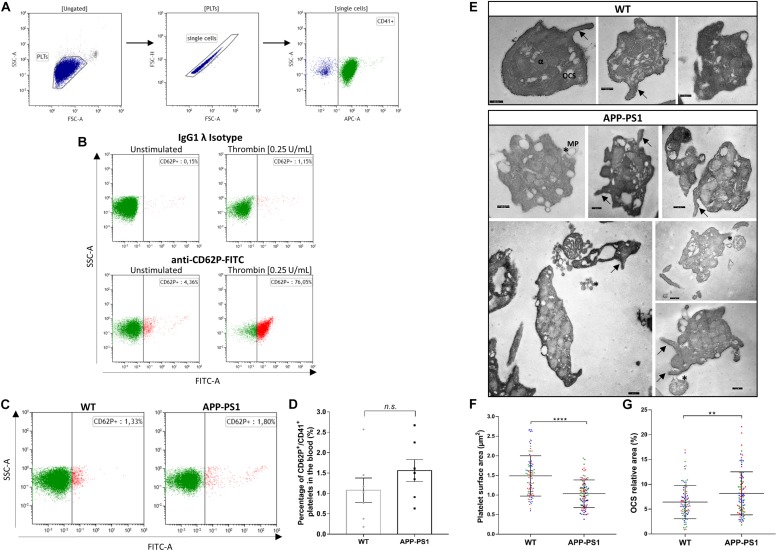
Activation status and ultrastructure analysis of peripheral blood platelets in APP-PS1 mice. Surface expression of CD62P by CD41^+^ platelets was assessed by flow cytometry to determine the activation status of blood-isolated platelets. Gating strategy for single CD41^+^ platelets **(A)**. Positive control for platelet activation: Unstimulated or thrombin-stimulated platelets were stained with CD62P or respective IgG isotype control antibody **(B)**. Both APP-PS1 and WT animals showed CD62P^+^/CD41^+^ platelets under unstimulated conditions **(C)**, but the percentage of activated platelets in the bloodstream of APP-PS1 and WT were not significantly different **(D)**. However, TEM analysis revealed ultrastructural differences between APP-PS1 and WT blood-isolated platelets. APP-PS1 platelets presented a more irregular cell membrane, with formation of membrane elongations (arrows), and released microparticles (asterisks, *), the last two being signs of platelet activation **(E)**. APP-PS1 platelets also exhibited a significantly smaller surface area **(F)** and an enlarged OCS **(G)** compared with WT platelets. The annotated structures are α-granules (α), open canalicular system (OCS), and microparticule (MP). Scale: 250 nm **(E)**. Graph bars represent mean ± SEM. Statistical analysis was performed by unpaired Student’s *t* test (**B:** *n* = 7/group; including three females and four males per group) and Mann–Whitney test **(F** and **G:** *n* = 90 platelets/genotype, including WT: three females and APP-PS1: two females and one male, with the 30 platelets analyzed per animal being color coded**)**. ****p < 0.0001; **p < 0.01.

The presence of platelets in brains with an AD amyloid pathology raises various questions such as their molecular profile and identity, their activation status, and their functional role in the brain parenchyma. As a first urging question, we addressed whether platelets are activated once they enter the brain parenchyma or whether they are already activated in the bloodstream. To that, we used FC to analyze the surface expression of CD62P, a widely used marker for platelet activation, as well as TEM for ultrastructural analysis of platelets isolated from the blood of APP-PS1 and WT mice. To assess CD62P expression by FC, we first gated the platelet population based on its pattern for forward and side light scattering and CD41 expression, and further defined the activated platelet population as CD62P^+^/CD41^+^ (Figures 2A,B). Up to 70% of CD41^+^ platelets expressed CD62P when stimulated *ex vivo* with thrombin as a positive control, and only very few CD62P^+^/CD41^+^ platelets were detected under native conditions (Figure 2B) indicating that the vast majority of platelets in the blood are not activated. Quantitative analysis revealed only a slight, but not significant, difference between the percentage of activated platelets in the bloodstream of APP-PS1 compared to WT mice (Figures 2C,D). TEM ultrastructural analysis (Figure 2E) revealed that WT platelets presented a round or discoid shape with smooth plasma membrane, although some WT platelets also showed small elongations and protrusions of the membrane, most likely being pseudopods. In APP-PS1 platelets, the plasma membrane was more irregular, with invaginations and protrusions. Some APP-PS1 platelets presented a microparticle formation (Figure 2E, asterisks), showing either microparticles under the process of budding off or already completely located in the extracellular space. Quantification of the platelet area showed that APP-PS1 platelets had an approximately 30% smaller surface in comparison with WT platelets (Figure 2F). Moreover, when compared to WT, APP-PS1 platelets presented an enlarged OCS (Figure 2G), a specific platelet organelle that appears as cytoplasmic vacuoles and thin tubular structures. In summary, our FC and TEM data suggests that APP-PS1 mice might possess a population of blood-circulating platelets exhibiting classical signs of activation, with a highly altered cell morphology and increased microparticle formation.

### Activated Platelets Are Present in the Brain of APP-PS1 Transgenic Mice

**FIGURE 3:**
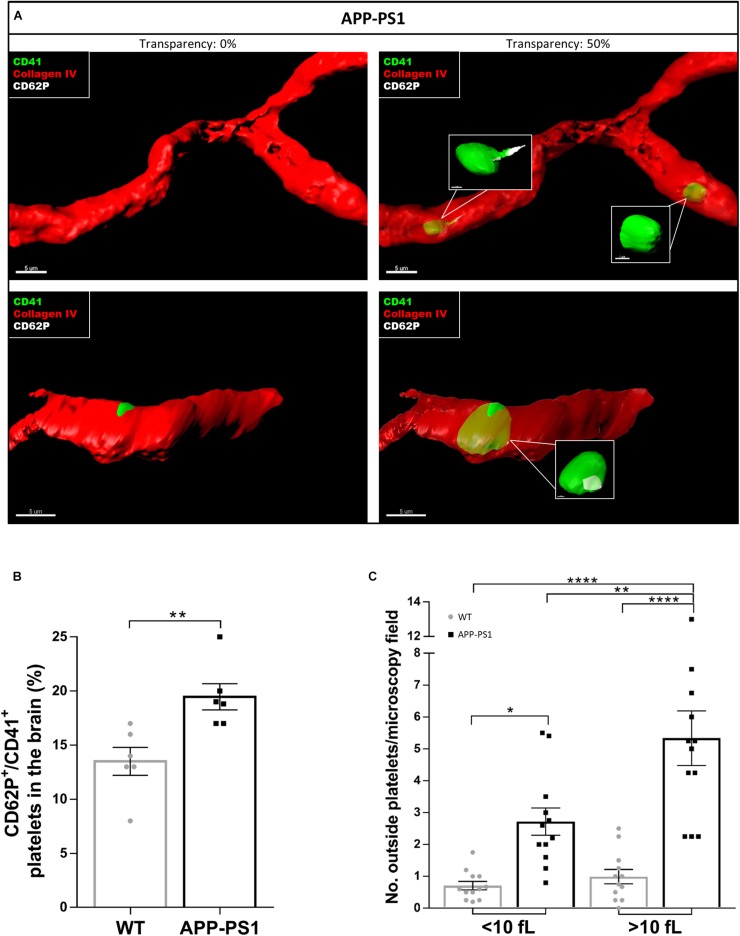
Activation status of platelets located in the brain of APP-PS1 mice. Immunohistochemical analysis revealed that CD41^+^ platelets (green) expressing the activation marker CD62P (white) circulate within cerebral blood vessels (red) in APP-PS1 mice **(A**, upper row**)**. Similarly, platelets in the process of fenestrating the vessel wall also expressed CD62P **(A**, lower row**)**. Quantitative analysis showed that APP-PS1 mice have a significantly higher percentage of CD62P^+^/CD41^+^ platelets in the brain compared to WT animals **(B)**. Moreover, in APP-PS1 mice, extravascular platelets exhibit a shift from single cells (<10 fL) to aggregates (>10 fL). Graph bars represent mean ± SEM (*n* = 6/group including WT: six females and APP-PS1: three females and three males). Statistical analysis was performed by unpaired Student’s *t* test **(B)** or two-way ANOVA with Tukey’s multiple comparison test **(C)**. *p < 0.05; **p < 0.01; and ****p < 0.0001. Scale: 5 μm **(A)** and 1 μm (**A**—zoom in).

Even though we took special care to avoid platelet activation during the isolation procedure, we cannot exclude the possibility that some of the analyzed activation hallmarks were artificially induced. Therefore, and to investigate the activation status of brain platelets, we stained non-perfused brain sections for CD41, CD62P, and Collagen IV. We further used the IMARIS software to characterize platelets located inside and outside the blood vessels in more detail regarding their CD62P expression. Using 3D models with different levels of transparency, we could allocate platelets inside or outside the blood vessels. By setting the transparency level of the reconstructed vessels at 0%, only platelets exposed to the outside of the vessel can be seen, whereas at 50% transparency, platelets are visible regardless of their location, i.e., inside or outside the vessel. As a result, some of the platelets within APP-PS1 and WT (not shown) cerebral blood vessels were CD62P^+^ and had cellular protrusions (Figure 3A), indicating the presence of activated platelets within the cerebral bloodstream. Similarly, platelets in the process of penetrating the vessel wall, i.e., partially visualized at 0% transparency and fully visible in the 50% transparent situation, expressed CD62P in WT and APP-PS1 mice (Figure 3A) indicating activation. At the quantitative level, the percentage of platelets that carry the activation marker CD62P was significantly higher in APP-PS1 compared to WT mice (Figure 3B). Taken together, these data suggest that platelets in the brain of APP-PS1 mice are activated.

A crucial hallmark of platelet function, at least in homeostasis, is platelet aggregation. Therefore, we raised the question whether platelets present in the brain parenchyma of APP-PS1 and WT mice might be aggregated. We used the IMARIS software to measure the volume of 3D modeled CD41^+^ structures. We further categorized these structures as single platelets when the volume was smaller than 10 fL or as platelet aggregates for volumes bigger than 10 fL. This threshold was set accordingly to the current knowledge on platelet size (Everds, 2004). In the brain of APP-PS1 mice, where a higher number of platelets were present, there was a clear shift from single platelets to small aggregates (Figure 3C).

### Platelets in the Brain Parenchyma Tightly Associate With Astrocytes

**FIGURE 4:**
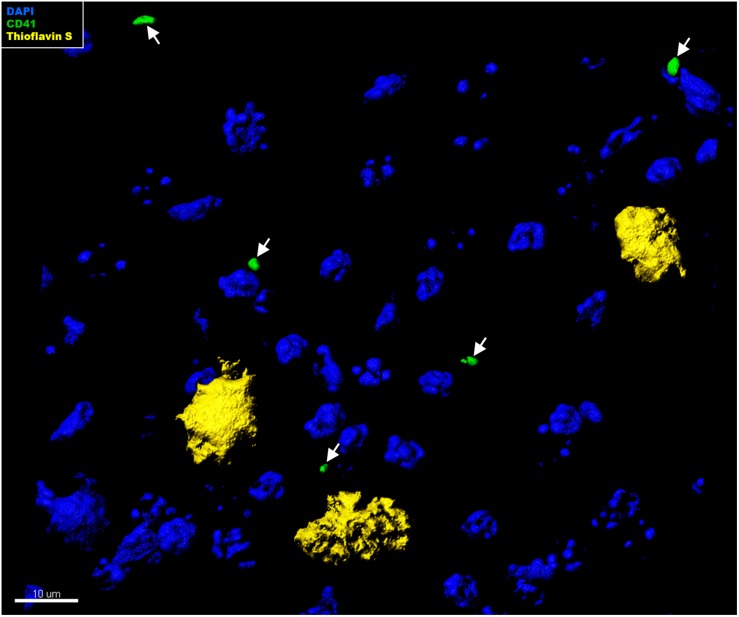
Immunohistochemical analysis of platelet interaction with amyloid plaques in the brain of APP-PS1 mice. Brain sections from APP-PS1 mice (*n* = 1/gender) were co-stained for platelets (green) and Thioflavin S (yellow). DAPI (blue) was used as nucleus stain. Representative confocal images were 3D modeled for co-localization analysis. In APP-PS1 mice, CD41^+^ platelets were not associated with Thioflavin S^+^ amyloid plaques.

**FIGURE 5:**
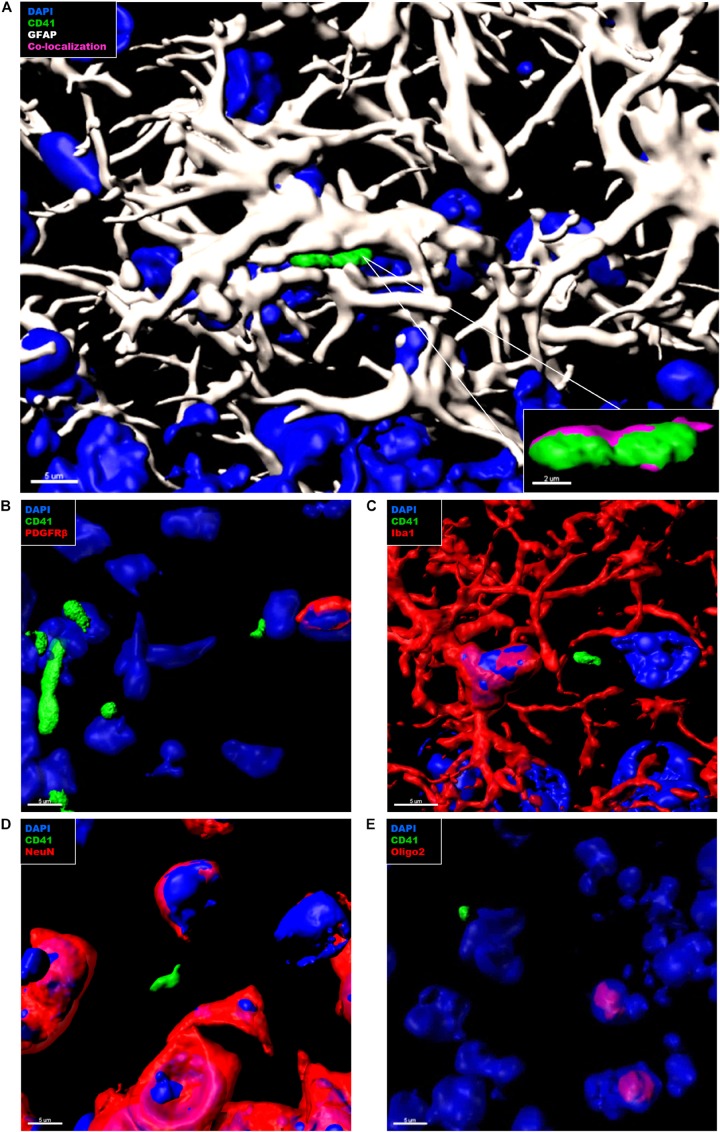
Immunohistochemical analysis of platelet interaction with neurovascular niche cellular components in the brain of APP-PS1 mice. Brain sections from APP-PS1 mice (*n* = 3, including two females and one male) were co-stained for platelets (CD41), **(A–E**, green**)**, and one of the following cell types: astrocytes (GFAP) **(A**, white**)**; pericytes (PDGFRβ) **(B**, red**)**, microglia (Iba-1) **(C**, red**)**, neurons (NeuN) **(D**, red**)**, or oligodendrocytes (Oligo2) **(E**, red**)**. DAPI (blue) was used as nucleus stain. Representative confocal images were selected for 3D surface projection and co-localization analysis. In APP-PS1 mice, platelets were found in tight association with astrocytes **(A)**. Co-localization of CD41 and GFAP signals is depicted in magenta **(A**, zoom in**)**. No further interactions were observed between CD41^+^ platelets and other immunolabeled cells **(B)**. Scale: 5 μm **(A,B)**, 2 μm **(B**—zoom in**)**.

In the context of CAA, platelets have been shown to accumulate at cerebrovascular Aβ deposits, where they seem to fuel Aβ deposition (Gowert et al., 2014; Kniewallner et al., 2015, 2016). To assess whether intra-parenchymal platelets might promote similar effects, we analyzed the localization of CD41^+^ platelets within the brain with respect to Thioflavin S^+^ amyloid plaques. However, an association between platelets and amyloid plaques in the brain of APP-PS1 mice was not observed (Figure 4). We further investigated the putative cellular interaction partners within the brain, assessing potential interactions with astrocytes, pericytes, microglia, neurons, and oligodendrocytes. In APP-PS1 mice (Figure 5), and similarly in WT animals (data not shown), brain-located CD41^+^ platelets were found in tight association with GFAP^+^ astrocytes, as revealed by 3D surface rendering and co-localization analysis of GFAP and CD41 signals showing astrocyte processes covering platelet surfaces (Figure 5A, magenta showing co-localization, and Supplementary Videos). No interactions were detected with PDGFRβ^+^ pericytes (Figure 5B), Iba1^+^ microglia (Figure 5C), NeuN^+^ neurons (Figure 5D), or Oligo2^+^ oligodendrocytes (Figure 5E). Approximately 60% of the platelets in the brain of APP-PS1 and WT mice were associated with astrocytes, whereas the other 40% did not associate with any other cell type analyzed (59.91 ± 5.76% vs. 40.08 ± 5.76%; *p* = 0.0352).

## Discussion

The current work reveals the presence of a significantly higher number of platelets in the brain parenchyma of APP-PS1 compared to WT mice. Platelets in the brain were more likely to be activated compared to the blood-circulating platelets, regardless of the genotype. In APP-PS1 mouse brains, the relative amount of activated platelets was significantly higher compared to WT. Platelets presumably invaded the brain and were found in tight association with astrocytes suggesting that these two cell types might interact, and that this interaction might contribute and shape AD pathology.

There is increasing evidence for blood-born elements influencing molecular, cellular, structural, and functional features of the brain in aging and in de- and regeneration in CNS diseases such as AD (Villeda et al., 2011, 2014; Middeldorp et al., 2016; Smith et al., 2018). In this context, blood components of emerging interest are platelets. For example, platelets are actively involved in the onset and development of cerebral amyloid angiopathy (CAA), a vascular pathology common among AD patients, which is characterized by platelet-derived Aβ depositions (Gowert et al., 2014; Jarre et al., 2014; Kniewallner et al., 2016, 2018). Interestingly, injections of platelets isolated from APP-Swedish Dutch Iowa mice, which is an AD transgenic mouse model with CAA pathology, into healthy age-matched control mice caused vessel damage, whereas WT platelet injection failed to do so (Kniewallner et al., 2018). Thus, we hypothesized that platelets in AD are intrinsically altered or dysfunctional, actively contributing to vascular damages (Kniewallner et al., 2018). To determine whether this kind of “aggressive or invasive” phenotype described for APP-Swedish Dutch Iowa mice is a general phenomenon in AD, we investigated platelets in a CAA-independent AD transgenic mouse model, the APP-PS1 (Jankowsky et al., 2001, 2004; Liu et al., 2004).

Here, we show that APP-PS1 mice contained a significantly higher number of platelets in the brain parenchyma compared to age-matched WT controls. Approximately 20% of the platelets present in the APP-PS1 brain invaded the intraparenchymal space, whereas in WT animals, only approximately 6% of the platelets were found within the brain parenchyma. Interestingly, brain intraparenchymal platelets appeared as single platelets as well as small aggregates, with APP-PS1 mice presenting a higher number of aggregated platelets compared to WT animals. Therefore, as the quantitative analysis of platelet density presented in Figure 1 did not differentiate between single platelets or platelet aggregates due to methodological limitations, putative aggregates might have been counted as individual platelets leading ultimately to an underestimation of the overall platelet load of APP-PS1 brains. Whether platelets penetrate the brain parenchyma actively or passively is still an ongoing question. We previously demonstrated that penetration of APP-Swedish Dutch Iowa platelets into the brain parenchyma is accompanied by release of platelet-derived matrix metalloproteinases, which promotes blood vessel disruption (Kniewallner et al., 2018). A similar mechanism might mediate the invasion of individual platelets into the brain of animals with subarachnoid hemorrhage (Doczi et al., 1986; Friedrich et al., 2010). In this model, platelets enter the brain parenchyma via “platelet-sized holes” in the basement membrane, possibly originated by platelet-derived proteases such as metalloproteinases-2 and -9 (Friedrich et al., 2010). Alternatively, platelets in AD might invade the brain parenchyma through a disrupted blood–brain barrier (BBB), as at the age of analysis (14 months) APP-PS1 mice present a comprised BBB (Janota et al., 2015).

### The Fate and Function of Platelets in the Brain?

Besides the penetration of platelets through the vessel wall, crucial questions are: What is the fate of platelets once they enter the brain parenchyma? What is their functional role in the brain? Are platelets able to home back to the circulation, for example, after discharging their contents? The latter has recently been shown in a mouse model of systemic shock, where platelets entered the brain parenchyma, released pathogenic levels of serotonin, and thereafter returned to the bloodstream (Cloutier et al., 2018). Alternatively, they might become simply eliminated from the brain parenchyma through phagocytosis by, for example, microglia, perivascular-macrophages, or astrocytes. Nevertheless, platelets, as long as they are within the CNS parenchyma, might communicate with certain cell types, modulate their functions, and shape certain pathogenic processes.

Various CNS lesions, e.g., neurotrauma, subarachnoid hemorrhage, demyelination, or experimental autoimmune encephalomyelitis show extravascular platelets in the brain, which are linked to the onset of either beneficial or detrimental responses in the brain (Pluta and Amek, 2008; Friedrich et al., 2010; Hayon et al., 2013; Sotnikov et al., 2013; Au et al., 2014; Kazanis et al., 2015; Rivera et al., 2016). One possible function of platelets in the brain seems to be the impact on neuroinflammation. This has been demonstrated in the context of experimental autoimmune encephalomyelitis (Sotnikov et al., 2013) and chronic hypertension (Bhat et al., 2017), where platelets promote neuroinflammation. In the context of AD, APP-Swedish Dutch Iowa platelets recruit and activate microglia to the penetrated vessels, leading to enhanced levels of tumor-necrosis-factor-alpha and supporting an inflammatory environment (Kniewallner et al., 2018). In the present work, we show that intra-parenchymal platelets are in tight and exclusive association with astrocytes, and this interaction might influence the astrocyte responses in the context of neuroinflammation or plaque load. Platelet–astrocyte interaction was previously reported in experimental autoimmune encephalomyelitis, an established model of neuroinflammation, demyelination, and neurodegeneration (Sotnikov et al., 2013), and in an experimental chronic hypertension rat model (Bhat et al., 2017), the latter being a well-known predisposing factor for vascular dementia and AD (Birkenhäger and Staessen, 2006). In both cases, platelet–astrocyte interactions were associated with enhanced neuroinflammation and neuronal injury (Sotnikov et al., 2013; Bhat et al., 2017). Whether platelet–astrocyte interaction is also promoting neuroinflammation and neurodegeneration in AD remains speculative, and it is a matter of further investigations. Of course, platelet–astrocyte interaction might occur as part of a clearance strategy, and platelets might simply be phagocytosed by astrocytes. However, the platelet–astrocyte association was not exclusively observed in APP-PS1 mice but also in age-matched WT animals (data not shown). Nevertheless, the load of platelets and the number of activated platelets was higher in the brain of APP-PS1 mice, and therefore, the impact on astrocytes might be different at the quantitative level.

Platelets might not only have detrimental functions in the brain but also beneficial ones. For example, it has recently been demonstrated that the exercise-induced stimulatory effects on adult hippocampal neurogenesis are partially mediated through platelets (Leiter et al., 2019). In the context of stroke and of demyelinating lesions, platelet-derived factors promote neurogenesis and neuroprotection (Hayon et al., 2013; Au et al., 2014; Kazanis et al., 2015). A long-term perspective might be to exploit the beneficial effects and to inhibit the detrimental effects of platelets in a therapeutic context. This could be achieved through pharmacological means, through cell therapies such as infusion of regeneration-promoting platelets, or through the use of platelet-derived factors.

### Platelets Are Activated in the Brain of APP-PS1 Mice but Not in the Bloodstream

Increased platelet activation in the blood has been previously reported in AD patients and in animal models of AD (Sevush et al., 1998; Ciabattoni et al., 2007; Stellos et al., 2010; Jarre et al., 2014; Canobbio et al., 2015, 2016). In AD, the activation status of platelets correlates with the rate of cognitive decline (Stellos et al., 2010; Canobbio et al., 2015), indicating that platelet activation might be functionally related to AD pathogenesis. In the APP-PS1 mice, we observed only a slight but not significantly higher platelet activation status in the bloodstream. Nevertheless, at the ultrastructural level, APP-PS1 mouse platelets showed clear signs of activation in cell morphology, i.e., significantly smaller size, membrane protrusions, and microparticle release. As a hypothesis, in AD, platelets might be either primed or even activated in the by bloodstream and become fully activated once entering the brain parenchyma. Regardless, a more detailed understanding of platelet activation as well as their underlying molecular mechanisms, especially in the context of CNS pathologies, is required.

### Alterations in the OCS of APP-PS1 Mouse Platelets

Abnormalities in the internal membrane system of platelets in AD have been previously described (Zubenko et al., 1987a, b, Hajimohammadreza et al., 1990; Piletz et al., 1991). In AD patients, 10–25% of platelets are atypical, exhibiting an extensive system of membrane-bounded “trabeculated cisternae” (Zubenko et al., 1987a, b; Hajimohammadreza et al., 1990). In our ultrastructural characterization, we observed that in APP-PS1 mouse platelets, vacuolar and tubular structures occupy a significantly wider area of the cytoplasm in comparison to WT controls. We considered these structures as being part of the platelet OCS, a system of interconnected and tortuous membrane-bound channels that spans through the platelet interior (Rumbaut and Thiagarajan, 2010; White, 2013; Selvadurai and Hamilton, 2018). Further analysis using specific dyes for the OCS, such as ruthenium red (Heijnen and Korporaal, 2017; Selvadurai and Hamilton, 2018), or morphological characterization of platelets using 3D electron microscopy are needed to determine whether the structures we observed are, indeed, part of the platelet OCS. Interestingly, abnormalities in platelet ultrastructure are common to other neurological disorders such as Parkinson’s disease, where platelets display an enlarged OCS (Factor et al., 1994), amyotrophic lateral sclerosis (Shrivastava et al., 2011), or major depression (Mendoza-Sotelo et al., 2010). Therefore, it is important to understand whether alterations in the platelet ultrastructure might directly affect the CNS environment and, in particular, to discern whether and how the enlargement of platelet OCS can be related with the pathological processes occurring in AD.

### Platelets in the Aging Brain

Even though most of the effects that we observed in the present study were more pronounced in APP-PS1 mice, they were also detected at a lower level in 14-month-old WT mice. Therefore, it will be crucial in future experiments to dissect the aging from the disease-specific effects. It is already well-known that platelet activity and function are altered with advanced age in humans as well as in mice (Zahavi et al., 1980; Kasjanovova and Balaz, 1986; Bastyr et al., 1990; Gleerup and Winther, 1995; Cowman et al., 2015; Jones, 2016). Nevertheless, since aging is the main risk factor for AD and various other neurodegenerative diseases, platelets might be a very promising target for the future development of therapeutic strategies to prevent brain aging and to prevent or treat age-related neurodegenerative diseases.

## Conclusion

Platelets are an emerging topic in brain aging and neurodegenerative diseases. The functional role of platelets in the brain and the underlying molecular and cellular mechanisms remain elusive, but platelets might have beneficial as well as detrimental functions in the brain. This requires further investigations, especially since platelets are a very promising and feasible target for cellular and pharmacological interventions, and targeting platelets might be a therapeutic approach to repair, regenerate, or even rejuvenate the aged and diseased brain.

## Data Availability Statement

The datasets generated for this study are available on request to the corresponding author.

## Ethics Statement

The animal study was reviewed and approved by BMWFW.

## Author Contributions

KK and DS designed the study, performed the experiments, and wrote the manuscript. MU designed the experiments and wrote the manuscript. HM designed and performed the experiments. LA designed the study and wrote the manuscript.

## Conflict of Interest

The authors declare that the research was conducted in the absence of any commercial or financial relationships that could be construed as a potential conflict of interest.

## Footnotes

1 http://www.jax.org/strain/005864

## Supplementary Material

VIDEO S1 3D animation created by surface rendering upon immunohistochemical stained APP-PS1 brain tissue showing CD41^+^ platelets (green) in tight association with GFAP^+^ astrocyte processes (white). Cell nuclei can be visualized in blue (DAPI staining).

VIDEO S2 3D animation created by surface rendering upon immunohistochemical stained WT brain tissue showing CD41^+^ platelets (green) in tight association with GFAP^+^ astrocyte processes (white). Cell nuclei can be visualized in blue (DAPI staining).

## References

[B1] Arman M. Holly P. Ponomaryov T. Brill A. (2015). “Role of platelets in inflammation,” in *The Non-Thrombotic Role of Platelets in Health and Disease*, eds Kerrigan S. W. Moran N. (London: Intechopen).

[B2] Au A. E. L. Sashindranath M. Borg R. J. Kleifeld O. Andrews R. K. Gardiner E. E. (2014). Activated platelets rescue apoptotic cells via paracrine activation of EGFR and DNA-dependent protein kinase. *Cell Death Dis.* 5:e1410. 10.1038/cddis.2014.373 25210793 PMC4540201

[B3] Bastyr E. J. Kadrofske M. M. Vinik A. I. (1990). Platelet activity and phosphoinositide turnover increase with advancing age. *Am. J. Med.* 88 601–606. 10.1016/0002-9343(90)90525-i 2161185

[B4] Bhat S. A. Goel R. Shukla R. Hanif K. (2017). Platelet CD40L induces activation of astrocytes and microglia in hypertension. *Brain Behav. Immun.* 59 173–189. 10.1016/j.bbi.2016.09.021 27658543

[B5] Birkenhäger W. H. Staessen J. A. (2006). Progress in cardiovascular diseases: cognitive function in essential hypertension. *Prog. Cardiovasc. Dis.* 49 1–10. 16867845 10.1016/j.pcad.2006.03.001

[B6] Canobbio I. Abubaker A. A. Visconte C. Torti M. Pula G. (2015). Role of amyloid peptides in vascular dysfunction and platelet dysregulation in Alzheimer’s disease. *Front. Cell. Neurosci.* 9:65. 10.3389/fncel.2015.00065 25784858 PMC4347625

[B7] Canobbio I. Visconte C. Oliviero B. Guidetti G. Zara M. Pula G. (2016). Increased platelet adhesion and thrombus formation in a mouse model of Alzheimer’s disease. *Cell. Signall.* 28 1863–1871. 10.1016/j.cellsig.2016.08.017 27593518

[B8] Ciabattoni G. Porreca E. Di Febbo C. Di Iorio A. Paganelli R. Bucciarelli T. (2007). Determinants of platelet activation in Alzheimer’s disease. *Neurobiol. Aging* 28 336–342. 16442186 10.1016/j.neurobiolaging.2005.12.011

[B9] Cloutier N. Allaeys I. Marcoux G. Machlus K. R. Mailhot B. Zufferey A. (2018). Platelets release pathogenic serotonin and return to circulation after immune complex-mediated sequestration. *Proc. Natl. Acad. Sci. U.S.A.* 115 E1550–E1559. 29386381 10.1073/pnas.1720553115 PMC5816207

[B10] Cowman J. Dunne E. Oglesby I. Byrne B. Ralph A. Voisin B. (2015). Age-related changes in platelet function are more profound in women than in men. *Sci. Rep.* 5 12235–12235. 26179119 10.1038/srep12235 PMC4503960

[B11] De Strooper B. Karran E. (2016). The cellular phase of Alzheimer’s disease. *Cell* 164 603–615. 26871627 10.1016/j.cell.2015.12.056

[B12] Doczi T. Joo F. Adam G. Bozoky B. Szerdahelyi P. (1986). Blood-brain barrier damage during the acute stage of subarachnoid hemorrhage, as exemplified by a new animal model. *Neurosurgery* 18 733–739. 10.1227/00006123-198606000-00010 3736802

[B13] Donner L. Falker K. Gremer L. Klinker S. Pagani G. Ljungberg L. U. (2016). Platelets contribute to amyloid-beta aggregation in cerebral vessels through integrin alphaIIbbeta3-induced outside-in signaling and clusterin release. *Sci. Signal.* 9:ra52 10.1126/scisignal.aaf6240 27221710

[B14] Everds N. (2004). “Chapter 17 – Hematology of the mouse,” in *The Laboratory Mouse*, ed. Bullock G. (London: Academic Press), 271–286. 10.1016/b978-012336425-8/50070-4

[B15] Evin G. Zhu A. Holsinger R. M. D. Masters C. L. Li Q.-X. (2003). Proteolytic processing of the Alzheimer’s disease amyloid precursor protein in brain and platelets. *J. Neurosci. Res.* 74 386–392. 14598315 10.1002/jnr.10745

[B16] Factor S. A. Ortof E. Dentinger M. P. Mankes R. Barron K. D. (1994). Platelet morphology in Parkinson’s disease: an electron microscopic study. *J. Neurol. Sci.* 122 84–89. 10.1016/0022-510x(94)90056-6 8195808

[B17] Friedrich V. Flores R. Muller A. Sehba F. A. (2010). Escape of intraluminal platelets into brain parenchyma after subarachnoid hemorrhage. *Neuroscience* 165 968–975. 10.1016/j.neuroscience.2009.10.038 19861151 PMC2814884

[B18] Frozza R. L. Lourenco M. V. De Felice F. G. (2018). Challenges for Alzheimer’s disease therapy: insights from novel mechanisms beyond memory defects. *Front. Neurosci.* 12:37. 10.3389/fnins.2018.00037 29467605 PMC5808215

[B19] Gleerup G. Winther K. (1995). The effect of ageing on platelet function and fibrinolytic activity. *Angiology* 46 715–718. 10.1177/000331979504600810 7639418

[B20] Gowert N. S. Donner L. Chatterjee M. Eisele Y. S. Towhid S. T. Münzer P. (2014). Blood platelets in the progression of Alzheimer’s disease. *PLoS ONE* 9:e90523. 10.1371/journal.pone.0090523 24587388 PMC3938776

[B21] Hajimohammadreza I. Brammer M. J. Eagger S. Burns A. Levy R. (1990). Platelet and erythrocyte membrane changes in Alzheimer’s disease. *Biochim. Biophys. Acta (BBA) – Biomembranes* 1025 208–214. 10.1016/0005-2736(90)90099-a 2142000

[B22] Hayon Y. Dashevsky O. Shai E. Varon D. Leker R. R. (2013). Platelet lysates stimulate angiogenesis, neurogenesis and neuroprotection after stroke. *Thrombosis Haemostasis* 110 323–330. 10.1160/th12-11-0875 23765126

[B23] Heijnen H. F. G. Korporaal S. J. A. (2017). “Platelet morphology and ultrastructure,” in *Platelets in Thrombotic and Non-Thrombotic Disorders: Pathophysiology, Pharmacology and Therapeutics: an Update*, eds Gresele P. Kleiman N. S. Lopez J. A. Page C. P. (Cham: Springer International Publishing), 21–37. 10.1007/978-3-319-47462-5_3

[B24] Humpel C. (2017). Platelets: their potential contribution to the generation of beta-amyloid plaques in Alzheimer’s disease. *Curr. Neurovasc. Res.* 14 290–298. 28677497 10.2174/1567202614666170705150535

[B25] International Alzheimer’s Disease [IAD] (2015). *World Alzheimer Report 2015: The Global Impact of Dementia.* London: Alzheimer’s Disease International.

[B26] Jankowsky J. L. Fadale D. J. Anderson J. Xu G. M. Gonzales V. Jenkins N. A. (2004). Mutant presenilins specifically elevate the levels of the 42 residue β-amyloid peptide in vivo: evidence for augmentation of a 42-specific γ secretase. *Hum. Mol. Genet.* 13 159–170. 10.1093/hmg/ddh019 14645205

[B27] Jankowsky J. L. Slunt H. H. Ratovitski T. Jenkins N. A. Copeland N. G. Borchelt D. R. (2001). Co-expression of multiple transgenes in mouse CNS: a comparison of strategies. *Biomol. Eng.* 17 157–165. 10.1016/s1389-0344(01)00067-3 11337275

[B28] Janota C. S. Brites D. Lemere C. A. Brito M. A. (2015). Glio-vascular changes during ageing in wild-type and Alzheimer’s disease-like APP/PS1 mice. *Brain Res.* 1620 153–168. 10.1016/j.brainres.2015.04.056 25966615 PMC4549169

[B29] Jarre A. Gowert N. S. Donner L. Munzer P. Klier M. Borst O. (2014). Pre-activated blood platelets and a pro-thrombotic phenotype in APP23 mice modeling Alzheimer’s disease. *Cell. Signall.* 26 2040–2050. 10.1016/j.cellsig.2014.05.019 24928203

[B30] Jones C. I. (2016). Platelet function and ageing. *Mamm. Genome* 27 358–366. 10.1007/s00335-016-9629-8 27068925 PMC4935731

[B31] Kasjanovova D. Balaz V. (1986). Age-related changes in human platelet function in vitro. *Mechan. Ageing Dev.* 37 175–182. 10.1016/0047-6374(86)90074-6 3821196

[B32] Kazanis I. Feichtner M. Lange S. Rotheneichner P. Hainzl S. Öller M. (2015). Lesion-induced accumulation of platelets promotes survival of adult neural stem/progenitor cells. *Exp. Neurol.* 269 75–89. 10.1016/j.expneurol.2015.03.018 25819103

[B33] Kniewallner K. M. Ehrlich D. Kiefer A. Marksteiner J. Humpel C. (2015). Platelets in the Alzheimer’s disease brain: do they play a role in cerebral amyloid angiopathy? *Curr. Neurovasc. Res.* 12 4–14. 10.2174/1567202612666150102124703 25557380 PMC4442621

[B34] Kniewallner K. M. Foidl B. M. Humpel C. (2018). Platelets isolated from an Alzheimer mouse damage healthy cortical vessels and cause inflammation in an organotypic ex vivo brain slice model. *Sci. Rep.* 8:15483. 10.1038/s41598-018-33768-2 PMC6195547 30341392

[B35] Kniewallner K. M. Wenzel D. Humpel C. (2016). Thiazine Red(+) platelet inclusions in Cerebral Blood Vessels are first signs in an Alzheimer’s disease mouse model. *Sci. Rep.* 6:28447. 10.1038/srep28447 PMC4921929 27345467

[B36] Lalonde R. Kim H. D. Maxwell J. A. Fukuchi K. (2005). Exploratory activity and spatial learning in 12-month-old APP(695)SWE/co+PS1/DeltaE9 mice with amyloid plaques. *Neurosci. Lett.* 390 87–92. 10.1016/j.neulet.2005.08.028 16169151

[B37] Leiter O. Seidemann S. Overall R. W. Ramasz B. Rund N. Schallenberg S. (2019). Exercise-induced activated platelets increase adult hippocampal precursor proliferation and promote neuronal differentiation. *Stem Cell Rep.* 12 667–679. 10.1016/j.stemcr.2019.02.009 30905740 PMC6450435

[B38] Li Q.-X. Cappai R. Evin G. Tanner J. E. Gray C. W. (1998). Products of the Alzheimer’s disease amyloid precursor protein generated by,β-secretase are present in human platelets, and secreted upon degranulation. *Am. J. Alzheimer’s Dis.* 13 236–244. 10.1177/153331759801300504

[B39] Liu L. Herukka S.-K. Minkeviciene R. van Groen T. Tanila H. (2004). Longitudinal observation on CSF Aβ42 levels in young to middle-aged amyloid precursor protein/presenilin-1 doubly transgenic mice. *Neurobiol. Dis.* 17 516–523. 10.1016/j.nbd.2004.08.005 15571987

[B40] Marschallinger J. Sah A. Schmuckermair C. Unger M. Rotheneichner P. Kharitonova M. (2015). The L-type calcium channel Cav1.3 is required for proper hippocampal neurogenesis and cognitive functions. *Cell Calcium* 58 606–616. 10.1016/j.ceca.2015.09.007 26459417

[B41] Mendoza-Sotelo J. Torner C. Alvarado-Vásquez N. Lazo-Langner A. López G. Arango I. (2010). Ultrastructural changes and immunolocalization of P-selectin in platelets from patients with major depression. *Psychiatry Res.* 176 179–182. 10.1016/j.psychres.2009.07.021 20193966

[B42] Michael J. Marschallinger J. Aigner L. (2019). The leukotriene signaling pathway: a druggable target in Alzheimer’s disease. *Drug Discov. Today* 24 505–516. 10.1016/j.drudis.2018.09.008 30240876

[B43] Middeldorp J. Lehallier B. Villeda S. A. Miedema S. S. Evans E. Czirr E. (2016). Preclinical assessment of young blood plasma for Alzheimer disease. *JAMA Neurol.* 73 1325–1333. 27598869 10.1001/jamaneurol.2016.3185 PMC5172595

[B44] Piaceri I. Nacmias B. Sorbi S. (2013). Genetics of familial and sporadic Alzheimer’s disease. *Front. Biosci. (Elite Ed.)* 5 167–177. 10.2741/e605 23276979

[B45] Piletz J. E. Sarasua M. Whitehouse P. Chotani M. (1991). Intracellular membranes are more fluid in platelets of Alzheimer’s disease patients. *Neurobiol. Aging* 12 401–406. 10.1016/0197-4580(91)90064-q 1770973

[B46] Pluta R. Amek M. U. (2008). Brain ischemia and ischemic blood-brain barrier as etiological factors in sporadic Alzheimer’s disease. *Neuropsychiatr. Dis. Treat.* 4 855–864. 19183778 10.2147/ndt.s3739 PMC2626921

[B47] Rivera F. J. Kazanis I. Ghevaert C. Aigner L. (2016). Beyond clotting: a role of platelets in CNS repair? *Front. Cell. Neurosci.* 9:511. 10.3389/fncel.2015.00511 26834562 PMC4718976

[B48] Rumbaut R. E. Thiagarajan P. (2010). “Chapter 2: General characteristics of platelets,” in *Platelet-Vessel Wall Interactions in Hemostasis and Thrombosis* eds Granger D. N. Granger J. P. (San Rafael, CA: Morgan & Claypool Life Sciences). 21452436

[B49] Selvadurai M. V. Hamilton J. R. (2018). Structure and function of the open canalicular system – The platelet’s specialized internal membrane network. *Platelets* 29 319–325. 10.1080/09537104.2018.1431388 29442528

[B50] Sevush S. Jy W. Horstman L. L. Mao W. W. Kolodny L. Ahn Y. S. (1998). Platelet activation in Alzheimer disease. *Arch. Neurol.* 55 530–536. 9561982 10.1001/archneur.55.4.530

[B51] Shrivastava M. Das T. K. Behari M. Pati U. Vivekanandhan S. (2011). Ultrastructural variations in platelets and platelet mitochondria: a novel feature in amyotrophic lateral sclerosis. *Ultrastruct. Pathol.* 35 52–59. 10.3109/01913123.2010.541985 21299344

[B52] Smith L. K. White C. W. III Villeda S. A. (2018). The systemic environment: at the interface of aging and adult neurogenesis. *Cell Tissue Res.* 371 105–113. 10.1007/s00441-017-2715-8 29124393 PMC5748432

[B53] Sotnikov I. Veremeyko T. Starossom S. C. Barteneva N. Weiner H. L. Ponomarev. (2013). Platelets recognize brain-specific glycolipid structures, respond to neurovascular damage and promote neuroinflammation. *PLoS One* 8:e58979. 10.1371/journal.pone.0058979 23555611 PMC3608633

[B54] Stellos K. Panagiota V. Kögel A. Leyhe T. Gawaz M. Laske C. (2010). Predictive value of platelet activation for the rate of cognitive decline in Alzheimer’s disease patients. *J. Cereb. Blood Flow Metabol.* 30 1817–1820. 10.1038/jcbfm.2010.140 20717123 PMC3023934

[B55] Unger M. S. Marschallinger J. Kaindl J. Hofling C. Rossner S. Heneka M. T. (2016). Early changes in hippocampal neurogenesis in transgenic mouse models for Alzheimer’s disease. *Mol. Neurobiol.* 53 5796–5806. 10.1007/s12035-016-0018-9 27544234 PMC5012146

[B56] Unger M. S. Schernthaner P. Marschallinger J. Mrowetz H. Aigner L. (2018). Microglia prevent peripheral immune cell invasion and promote an anti-inflammatory environment in the brain of APP-PS1 transgenic mice. *J. Neuroinflammation* 15 274. 10.1186/s12974-018-1304-4 PMC6151006 30241479

[B57] Van Cauwenberghe C. Van Broeckhoven C. Sleegers K. (2015). The genetic landscape of Alzheimer disease: clinical implications and perspectives. *Genet. Med.* 18:421. 10.1038/gim.2015.117 26312828 PMC4857183

[B58] Villeda S. A. Luo J. Mosher K. I. Zou B. Britschgi M. Bieri G. (2011). The ageing systemic milieu negatively regulates neurogenesis and cognitive function. *Nature* 477 90–94. 10.1038/nature10357 21886162 PMC3170097

[B59] Villeda S. A. Plambeck K. E. Middeldorp J. Castellano J. M. Mosher K. I. Luo J. (2014). Young blood reverses age-related impairments in cognitive function and synaptic plasticity in mice. *Nat. Med.* 20 659–663. 10.1038/nm.3569 24793238 PMC4224436

[B60] White J. G. (2013). “Chapter 7 – Platelet structure,” in *Platelets*, Third Edn, ed. Michelson A. D. (Cambridge, MA: Academic Press), 117–144.

[B61] Yun S.-H. Sim E.-H. Goh R.-Y. Park J.-I. Han J.-Y. (2016). Platelet activation: the mechanisms and potential biomarkers. *BioMed. Res. Int.* 2016 9060143–9060143. 27403440 10.1155/2016/9060143 PMC4925965

[B62] Zahavi J. Jones N. A. G. Leyton J. Dubiel M. Kakkar V. V. (1980). Enhanced in vivo platelet “release reaction” in old healthy individuals. *Thrombosis Res.* 17 329–336. 10.1016/0049-3848(80)90067-5 6445093

[B63] Zubenko G. S. Cohen B. M. Boller F. Malinakova I. Keefe N. Chojnacki B. (1987a). Platelet membrane abnormality in Alzheimer’s disease. *Ann. Neurol.* 22 237–244. 10.1002/ana.410220208 3662454

[B64] Zubenko G. S. Malinakova I. Chojnacki B. (1987b). Proliferation of internal membranes in platelets from patients with Alzheimer’s disease. *J. Neuropathol. Exp. Neurol.* 46 407–418. 10.1097/00005072-198707000-00001 3598604

